# Investigation of the fermentation filtrate from soapberry (*Sapindus mukorossi* Gaertn.) pericarp on improving the microbial diversity and composition of the human scalp

**DOI:** 10.3389/fmicb.2024.1443767

**Published:** 2024-10-10

**Authors:** Chong Xu, Danyang Pan, Dexiang Zhang, Lin Lin, Yiti Chen, Shuangcheng Liang, Jingyu He

**Affiliations:** ^1^Research Center of New Material, Guangzhou Uniasia Cosmetic Technology Co., Ltd, Guangzhou, China; ^2^Research Center of Chinese Herbal Resource Science and Engineering, Guangzhou University of Chinese Medicine, Key Laboratory of Chinese Medicinal Resource from Lingnan (Guangzhou University of Chinese Medicine), Ministry of Education, Joint Laboratory of National Engineering Research Center for the Pharmaceutics of Traditional Chinese Medicines, Guangzhou, China; ^3^School of Chemistry, South China Normal University, Guangzhou, China

**Keywords:** microbial community, human scalp, *Sapindus mukorossi* Gaertn, fermentation filtrate, bacterial-fungal inter-kingdom networks

## Abstract

**Introduction:**

Microorganisms as a component of scalp ecosystem play a vital role in human scalp health. Soapberry pericarp is employed in improving scalp health, and its content of active ingredients could be enhanced resulted in fermentation. This study aims to investigate the effect of the fermentation filtrate from soapberry pericarp on the diversity of scalp microorganisms.

**Methods:**

The components in fermentation filtrate from soapberry pericarp were analyzed by HPLC-Q-Orbitrap HRMS, and 16S and ITS sequences of 198 samples from three different used stages (Day 0, Day 7, Day 28) were sequenced using the Illumina Novaseq platform. Microbial diversity was assessed using alpha diversity (Chao1 and Shannon indexes) and beta diversity (weighted unifrac and unweighted unifrac). Principal coordinate analysis (PCoA) and linear discriminant analysis (LDA) effect size analysis (LEfSe) were used to visualize microbial variation among different stages.

**Results:**

There were 22 components were identified in the fermentation filtrate from soapberry pericarp by HPLC-Q-Orbitrap HRMS. The alpha-diversity and beta-diversity analyses showed that scalp microbial diversity and composition were influenced by the fermentation filtrate of soapberry pericarp. Based on functional analysis, this study found an enrichment of healthy scalp-related bacterial pathways, such as amino acid, nucleoside, and nucleotide biosynthesis, while a decrease in fungal pathogenesis pathways, specifically saprotroph and symbiotroph pathways, was observed.

**Discussion:**

The study described about the complex community dynamics of human scalp microorganisms during the stages of using the fermentation filtrate from soapberry pericarp. This result will help rationally utilize the fermentation filtrate from soapberry pericarp to keep or improve human scalp health.

## Introduction

The human scalp microbiota is a complex ecosystem, including thousands of species of microorganisms such as bacteria, fungi, viruses, and mites (Oh et al., 2014; Chen et al., 2018). These microbial communities can secrete certain components to resist harmful microorganisms in order to maintain human scalp health. However, the human scalp can influence the composition of the microbiota by providing the required nutrients, such as sebum, sweat, and other substances. Over time, homeostatic scalp health is achieved via this crosstalk between the scalp and the microorganisms through a commensal relationship, which is a delicate balance. However, when this balance is disrupted, the scalp’s microbial community becomes disturbed, leading to hair or scalp conditions like pruritus and dandruff (Constantinou et al., 2021). Currently, the significance of scalp microorganisms in maintaining scalp health has garnered considerable attention and investigation. Metagenomic analyses have identified *Basidiomycota*, *Actinobacteriota,* and *Firmicutes* as the main fungal and core bacterial phyla found on healthy scalps, and the genera *Malassezia*, *Cutibacterium*, and *Staphylococcus* are further identified (Xu et al., 2016). Among the fungal microbiota, *Malassezia* is the most abundant fungus on the scalp, exhibiting a lipid-dependent and lipophilic lifestyle.

This lipophilic nature allows *Malassezia* to proliferate in regions with high sebum secretion, impacting the microecological environment of the scalp (Gioti et al., 2013; Saxena et al., 2021). Among the bacterial microbiota, *Cutibacterium* and *Staphylococcus* are the most abundant bacteria on the scalp. These bacteria exhibit mutual inhibition. *Cutibacterium* produces bacteriocins that suppress the growth of *Staphylococcus*, whereas *Staphylococcus* mediates the fermentation of glycerol, thereby inhibiting the overgrowth of *Cutibacterium* (Xu et al., 2016). Overall, scalp microorganisms can influence the scalp situation through both microbial-microbial and microbial-host interactions. However, few studies have explored effective strategies for maintaining a healthy balance of the human scalp.

*Sapindus mukorossi* Gaertn., also named soapberry, belongs to the family Sapindaceae. Soapberry has long been known for its detergent properties (Xue et al., 2022). It is usually made into soaps, detergents, shampoos, and other products that are widely used for daily washing. The Compendium of Materia Medica, an ancient Chinese medical book, recommends that soapberry pericarp be used daily to wash the hair to cure dandruff (Shizhen, 1975; Xu et al., 2021). Modern research shows that soapberry is rich in various bioactive compounds, such as saponins (0.98–13.26%), flavonoids (0.31–1.74%), amino acids, and vitamins (Yuanyuan et al., 2021). It also exhibits multiple pharmacological activities, including anti-inflammatory, anti-bacterial, anti-viral, and other bioactive properties (Xu et al., 2021). Saponins, the primary bioactive compounds, display excellent non-ionic surface activity, high foaming properties, and strong cleaning abilities, making soapberry a potential natural surfactant for eco-friendly detergents (Abhirup Basu et al., 2015; Xu et al., 2021). Therefore, soapberry is employed in scalp-related products to enhance scalp health and hygiene by cleansing the scalp, promoting scalp metabolism, and providing essential nutrients for hair growth.

Microbial fermentation has gained increasing popularity in recent years due to its advantages, such as the use of cheap raw materials, mild reaction conditions, minimal environmental pollution, and the high purity of natural products (Gao et al., 2019; Sun et al., 2023). This biotransformation technique has been extensively employed in the study of numerous active compounds. It works by breaking down large molecular weight substances through microbial metabolism, concentrating active compounds.

Additionally, it converts these substances into smaller, more active forms, enhancing detoxification and efficiency by utilizing various enzymes secreted by microorganisms to release active constituents from plant cell walls (Luan et al., 2021; Sun et al., 2022). Consequently, this method effectively reduces plant substances with toxic side effects and decreases irritability. Previous studies have shown that the soapberry pericarp water extract fermented with *Lactobacillus plantarum* can decompose macromolecular substances (e.g., polysaccharides) into small metabolites, thereby increasing saponin contents (Heng et al., 2015; Wan Kaibo et al., 2024). However, the effect of fermentation filtrates from soapberry pericarp on human scalp microorganisms has not yet been reported.

We conducted a 28-day study to investigate the influence of the fermentation filtrate from soapberry pericarp on human scalp microorganisms using a generation sequencing technique based on the 16S amplicons and fungal ITS.

Changes in the scalp microflora were compared before and after the use of the fermentation filtrate from soapberry pericarp, and their variations and biological functions were further analyzed. To the best of our knowledge, this is the first time that the scalp microbiome has been profiled during the use of the fermentation filtrate from soapberry pericarp.

## Materials and methods

### Materials and chemicals

Soapberry pericarp was collected from Guiping City, Guangxi, China, and was identified by Associate Prof. Menghua Liu, School of Pharmaceutical Sciences, Southern Medical University, Guangdong, China. *Lactiplantibacillus plantarum* (GDMCC1.140) was obtained from the Guangdong Microbial Culture Collection Center (Guangzhou, China). The voucher specimens were stored at the Research Center of New Material, Guangzhou Uniasia Cosmetic Technology Co., Ltd. 2,2-Diphenyl-1-picrylhydrazyl and ginsenoside Re were purchased from Shanghai Macklin Biochemical Technology Co., Ltd. (Shanghai, China). Acetonitrile (LC/MS grade) and formic acid (HPLC grade) were purchased from Fisher Scientific (Fair Lawn, NJ, USA). Water was obtained from an ultrapure water system (Purelab Plus, Pall, Port Washington, NY, USA). Other chemicals (AR) were purchased from Shanghai Aladdin Biochemical Technology Co., Ltd. (Shanghai, China).

### Preparation of the fermentation filtrate from soapberry pericarp

100 g of soapberry pericarp was extracted with 1 kg of pure water at 100°C for 1 h. After the filtration process, the residue was repeatedly extracted once. The filtrates were combined and concentrated at 60°C in a vacuum, yielding soapberry pericarp water extract equal to 10% (w/w) raw material. Subsequently, 500 g of the yielded soapberry pericarp water extract, 25 g of MRS medium, and 475 g of pure water were mixed, and the pH was adjusted to pH 7.0 before sterilization at 121°C for 15 min. Meanwhile, the *L. plantarum* strain in the glycerol tube was inoculated into MRS medium and activated at 37°C for 48 h. Then, the activated culture of *L. plantarum* was diluted to obtain an initial density of 10^7^ CFU/mL in the mixture mentioned above, and anaerobic fermentation was carried out at 37°C for 3 days. The fermentation filtrate from soapberry pericarp was obtained after centrifugation at 4°C for 30 min and stored at 4°C for further study.

### Saponin contents assay

Saponin contents in fermentation filtrate of soapberry pericarp and soapberry pericarp water extract were measured according to the determination method of total saponins under “Ginseng total saponins” extract recorded in the Chinese Pharmacopoeia (2020 edition) (Commission, 2020), namely the vanillin-concentrated sulfuric acid colorimetry spectrophotometry.

### DPPH radical scavenging activity assay

DPPH radical scavenging activity was measured according to a reported method (Li et al., 2020). Sample solution of different volumes (0–18 μL) was added to a 96-well plate and then adjusted to 100 μL with 50% ethanol. The sample mixture was incubated for 30 min at 25°C after adding a freshly prepared 95% ethanol solution of DPPH (200 μL; 80 μg/mL). The absorbance was measured at 519 nm. All determinations were performed in triplicate. In a control experiment, 50% ethanol (100 μL) was used. The radical scavenging capability of DPPH was calculated using the following equation: Scavenging effect (%) = (1 − A_sample_/A_control_) × 100, where A_sample_ and A_control_ are the absorbance of the sample and the control, respectively.

### UHPLC-Q-Orbitrap HRMS analysis

Thermo Vanquish UHPLC System (Thermo Fisher Scientific, USA) and a Thermo Scientific Hypersil GOLD C18 (2.1 i.d. ×100 mm, 1.9 μm, Thermo Fisher Scientific). The mobile phase was composed of 0.1% (v/v) formic acid solution (A) and acetonitrile (B). The following gradient elution program was used: 0–1 min, 5% B; 1–8 min, 5–40% B; 8–30 min, 40–95% B. The flow rate was kept at 0.30 mL/min. The injection volume was 5 μL, and the column temperature was set at 30°C. The parameters of the Orbitrap Fusion mass spectrometer system (Thermo Fisher Scientific, USA) were as follows: H-ESI electric spray ion source; positive and negative ion scanning; quadrubode ion monitoring mode; ion source temperature: 320°C; ion detection range (m/z): 100–2000; positive mode voltage: 3500 V; negative mode voltage: 2500 V; Orbitrap resolution: 60000.

### Volunteer recruitment

Volunteers with overall healthy physical conditions were recruited from the Guangzhou Uniasia Cosmetic Technology Co., Ltd., which holds a human efficacy laboratory. In our survey, 33 volunteers were recruited based on the following inclusion and exclusion criteria.

The inclusion criteria for the study were as follows: (1) participants aged between 18 and 60 years; (2) able to understand the experimental procedures, sign an informed consent form, and voluntarily participate in the study; (3) participants were required to complete the trial and maintain a consistent lifestyle throughout the study duration, with no additional use of similar products.

The exclusion criteria were: (1) use of antihistamines within the past week or immunosuppressive agents within the past month; (2) participation in other clinical research in the last 3 months; (3) known allergies or hypersensitivity to any component of the investigational product; (4) currently pregnant, lactating, or planning pregnancy; (5) history of cosmetic treatments within the last 3 months; (6) presence of serious systemic, autoimmune, or immunodeficiency diseases; and (7) history of skin treatment, cosmetic, or other interventions that could influence the test results.

All experiments were carried out in accordance with approved guidelines and regulations, and all volunteers signed informed consent forms that explained the procedure and purpose of the study.

### Treatment

The volunteers were asked to use the fermentation filtrate from soapberry pericarp three times a week for a period of 4 weeks. The treatment consisted of 20 min of scalp massage with 10 mL of fermentation broth or normal saline and then blow-dried with a hair dryer.

### Sample collection, DNA extraction, and PCR amplification

A sterile cotton swab was moistened with sterile normal saline, and the selected area of the head was swabbed for 30 s. Then, the swab was placed back into the collection tube immediately and flash-frozen with liquid nitrogen, stored at −80°C, and transported on dry ice to Novogene Company (Novogene, Beijing, China).

The total genomic DNA of the swap samples was extracted using the CTAB method. DNA quality was assessed using 1% agarose gel electrophoresis, and DNA concentration was determined by NanoDrop. According to the concentration, DNA was diluted to 1 ng/μL using sterile water. The 16S rRNA/ITS genes were amplified using the specific primers with barcodes. The V4 region of bacteria and the ITS1 region of fungi were selected for amplification and sequencing. The primers used for the 16S rDNA gene were 515F (5′-GTGCCAGCMGCCGCGGTAA-3′) and 806R (5′-GGACTACHVGGGTWTCTAAT-3′). The primers for the ITS1 rDNA gene were ITS1-5F (5′-GGAAGTAAAAGTCGTAACAAGG-3′) and ITS1R (5′-GCTGCGTTCTTCATCGATGC-3′). PCR reaction mixture contained 2 μM forward and reverse primers, 15 μL Phusion^®^ High-Fidelity PCR Master Mix (New England Biolabs), and about 10 ng template DNA. The thermocycling conditions were as follows: initial denaturation at 98°C for 1 min, followed by 30 cycles of denaturation at 98°C for 10 s, annealing at 50°C for 30 s, and elongation at 72°C for 30 s. Finally, 72°C for 5 min.

### Library preparation and sequencing

Library construction was performed using the TruSeq^®^ DNA PCR-Free Sample Preparation Kit (Illumina, USA). Then, the constructed libraries were quantified by Qubit and Q-PCR and sequenced by NovaSeq PE250 (Illumina).

### Bioinformatics and data analysis

Raw data were generated by Illumina and truncated by removing the barcode and primer sequence. Then, the R1 and R2 reads were joined using the Flash software (V1.2.11)^1^ (Magoc and Salzberg, 2011), and the obtained splicing sequence was filtered and analyzed through Fastp software (Version 0.23.1) to obtain high-quality clean tags (Bokulich et al., 2013). Subsequently, the chimera sequences were removed, and the effective tags were obtained using the vsearch (V2.16.0) package (Edgar et al., 2011).

All the effective tags were clustered into operational taxonomic units (OTUs) using the Uparse software (Uparse v7.0.1001)^2^ (Edgar, 2013) with 97% identity, and the representative sequence of OTUs (with the highest frequency) was selected for further annotation. The Silva Database^3^ (Quast et al., 2013) and the Unite Database^4^ (Herr et al., 2015) were used to annotate taxonomic information for the bacterial 16S rRNA and fungal ITS, respectively.

The Qiime software (Version 1.9.1) was performed to calculate the alpha diversity index (such as Chao 1 and Shannon) and the beta diversity index (such as weighted unifrac and unweighted unifrac). For the alpha diversity index, the differences between groups were analyzed using the Kruskal-Wallis H test for those with uneven variance, which was achieved through a medical statistical module in Home for Researchers.^5^ For the beta diversity index, the Bray–Curtis dissimilarity matrices were calculated (Bray and Curtis, 1957), and then the statistically significant differences were determined using permutational multivariate analyses of variance (PERMANOVA) on a cloud platform.^6^ The linear discriminant analysis (LDA) effect size analysis (LEfSe) was employed to assess significant taxonomic differences in bacteria and fungi. Using the Kruskal-Wallis rank-sum test (*α* = 0.05) to identify groups exhibiting significantly different abundances across categories, followed by a logarithmic LDA score (threshold = 4.0 for bacteria and threshold = 3.0 for fungi) to estimate the effect size of each discriminant feature, all steps were achieved through R scripts in R software (Version 4.3.1). In addition, the principal coordinate analysis (PCoA) graphs and linear discriminant analysis (LDA) effect size analysis (LEfSe) graphs were plotted using R software (Version 4.3.1).

In all analyses, a *p-*value of <0.05 was set to indicate statistical significance.

## Results

### Saponin contents and DPPH antioxidant activity analyses of the fermentation filtrate from soapberry pericarp and soapberry pericarp water extract

**Figure 1:**
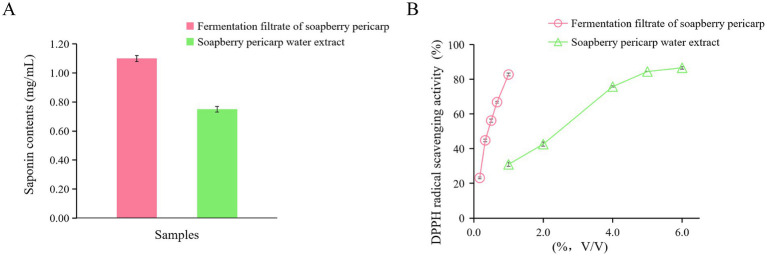
Saponin contents and DPPH antioxidant activities of the fermentation filtrate from soapberry pericarp and soapberry pericarp water extract. **(A)** saponin contents. **(B)** DPPH radical scavenging activities.

In order to determine changes in major chemicals after fermentation with *L. plantarum*, the saponin content in the fermentation filtrate from soapberry pericarp and soapberry pericarp water extract was studied. The results presented in Figure 1A indicate that saponin contents in the fermentation filtrate from soapberry pericarp and soapberry pericarp water extract were 1.10 ± 0.02 mg/mL and 0.75 ± 0.02 mg/mL, respectively. Notably, the saponin contents in the fermentation filtrate from soapberry pericarp exhibited a 46.67% increase compared to that in the soapberry pericarp water extract.

To evaluate the antioxidant activities of the fermentation filtrate from soapberry pericarp and the soapberry pericarp water extract, DPPH radical scavenging activity measurements were conducted (Figure 1B). The results indicated that the fermentation filtrate from soapberry pericarp had a stronger DPPH radical scavenging activity than that of soapberry pericarp water extract below 1.0% (V/V). Antioxidant activity, as determined by IC_50_ values, is defined as the concentration of the antioxidant required to achieve 50% scavenging of the radicals. The IC_50_ values for the fermentation filtrate from soapberry pericarp and the soapberry pericarp water extract were 0.472 and 2.44%, respectively, for DPPH radical scavenging.

### UHPLC-Q-Orbitrap HRMS analysis of fermentation filtrate of soapberry pericarp

Based on the above results, the fermentation filtrate from soapberry pericarp has advantages over soapberry pericarp water extract. Therefore, the compounds of the fermentation filtrate from soapberry pericarp were further studied. The analysis result of HPLC-Q-Orbitrap HRMS, as detailed in Table 1, shows that 22 components were identified in the fermentation filtrate from soapberry pericarp. Combining with the precursor ions and MS/MS spectrum data, two major chemical types were found. Compounds 4, 5, 6, 7, 8, 9, 10, 11, and 12 were identified as glycolipids, and compounds 13, 14, 15, 16, 17, and 18 were identified as triterpenoid saponins. The other seven compounds were sucrose (1) and its acetylated sucrose (2), 2-methyl benzoic acid (3), lauroyl sarcosine (19), folipastatin (21), and two long-chain fatty acids, including15-hydroxy eicosanoid acid (20) and stearic acid (22).

**Table 1:** The compounds identified in the fermentation filtrate from soapberry pericarp by UHPLC-Q-Orbitrap HRMS.

Compound	Formula	Precursor ions	*m/z* (error, ppm)	MS/MS *m/z* (error, ppm)	Identification
1	C_12_H_22_O_11_	[M + HCOO]-	387.11316 (−0.15)	341.10779 (−0.04) [M-H]-	Sucrose
2	C_14_H_24_O_12_	[M + HCOO]-	429.12308 (−0.81)	383.11835 (−0.05) [M-H]-	O-Acetylsucrose
3	C_8_H_8_O_2_	[M-H]-	135.04413 (0.07)	120.02033 (−0.25) [M-H-CH_3_]-	2-Methylbenzoic Acid
4	C_45_H_76_O_24_	[M + HCOO]-	1045.46594 (2.051)	999.46161 (−2.66) [M-H]-	Mukurozioside Ib
5	C_51_H_86_O_28_	[M + HCOO]-	1193.52026 (−7.41)	1145.51648 (6.03) [M-H]-	Mukurozioside IIa
6	C_45_H_78_O_24_	[M + HCOO]-	1047.48193 (−3.47)	1001.47687 (−3.02) [M-H]-	Mukurozioside Ia
7	C_31_H_42_O_10_	[M + HCOO]-	619.27002 (−4.88)	573.26447 (−4.95) [M-H]-	Papulacandin D
8	C_47_H_80_O_25_	[M + HCOO]-	1089.49170 (−4.23)	1043.48743 (−3.06) [M-H]-	Mukurozioside IId or isomer
9	C_47_H_80_O_25_	[M + HCOO]-	1089.49121 (−4.47)	1043.48706 (−3.43) [M-H]-	Mukurozioside IId or isomer
10	C_33_H_56_O_15_	[M + HCOO]-	737.35748 (−1.51)	691.35223 (−1.37) [M-H]-	Pyishiauoside Ib
11	C_47_H_80_O_25_	[M + HCOO]-	1089.49121 (−4.47)	1043.48669 (−3.85) [M-H]-	Mukurozioside IId or isomer
12	C_33_H_58_O_15_	[M + HCOO]-	739.37323 (1.47)	693.36755 (−1.64) [M-H]-	Capsoside A
13	C_46_H_74_O_16_	[M + HCOO]-	927.49304 (−0.92)	881.48773 (−1.58) [M-H]-	Sapindoside B
14	C_41_H_66_O_12_	[M + HCOO]-	795.44977 (−2.76)	749.44415 (−2.90) [M-H]-	Sapindoside A
15	C_48_H_76_O_17_	[M + HCOO]-	969.50220 (−3.15)	923.49890 (−0.97) [M-H]-	Rarasaponin II
16	C_48_H_76_O_17_	[M + HCOO]-	969.50269 (−2.66)	923.49902 (−0.85) [M-H]-	Rarasaponin III
17	C_50_H_78_O_18_	[M + HCOO]-	1011.51361 (−2.31)	965.50977 (−0.67) [M-H]-	Rarasaponin VI
18	C_50_H_78_O_18_	[M + HCOO]-	1011.51282 (−3.14)	965.50891 (−1.53) [M-H]-	Rarasaponin VI isomer
19	C_15_H_29_O_3_N	[M-H]-	270.20657 (0.20)	226.21684 (0.29) [M-H-CO_2_]-	Lauroyl sarcosine
20	C_20_H_40_O_3_	[2 M-H]-	655.44055 (−1.02)	327.28955 (0.17) [M-H]-	15-Hydroxyeicosanoic acid
21	C_23_H_24_O_5_	[M-H]-	379.1572 (3.2)	361.14798 (4.55) [M-H-H_2_O]-	Folipastatin
22	C_22_H_44_O_3_	[M-H]-	355.32065 (0.02)	309.31582 (0.68) [M-H-HCOOH]-	Stearic acid

### Diversity of the microbial community on the human scalp

**Figure 2:**
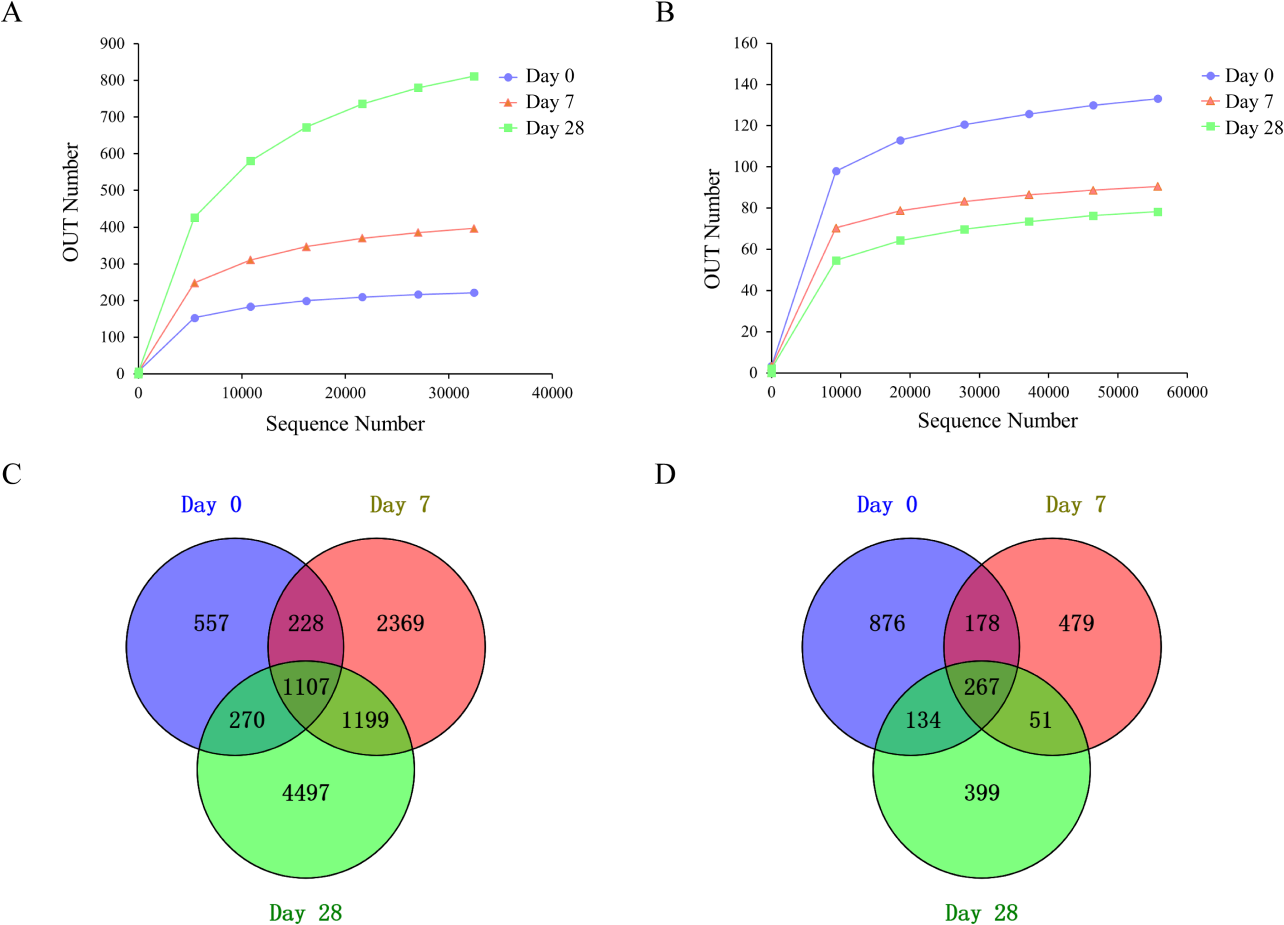
Rarefaction curves and Venn diagrams of the bacterial and fungal communities among the stages of Day 0, Day 7, and Day 28. **(A)** Rarefaction curves of the bacteria community; **(B)** rarefaction curves of the fungal community; **(C)** Venn diagrams of the bacteria community; **(D)** Venn diagrams of the fungal community.

This work aimed to evaluate the changes in the human scalp microbial community during the stages of using the fermentation filtrate from soapberry pericarp. A total of 22,431,905 sequences were produced through deep sequencing of the microbial community, with 22,226,919 sequences meeting quality control standards, representing 99.1% of the sequences acquired. There were 9,162,476 sequences obtained from bacteria and 13,064,443 sequences obtained from fungi. All of the effective sequences with ≥97% similarity were assigned to the same operational taxonomic unit (OUT). A total of 13,379 OTUs were obtained, including 10,868 bacteria and 2,511 fungi. The rarefaction curve tends to be flat (Figures 2A,B), indicating that the sequencing depth is sufficient.

Among all OTUs analyzed in this study, for bacteria, three stages, including Day 0, Day 7, and Day 28, shared 1,107 OTUs, mainly *Lactobacillus*, *Staphylococcus*, *Cutibacterium*, *Lawsonella*, *unidentified_Chloroplast*, *Bacteroides*, *Weissella*, *Vibrio*, and *Ralstonia* at the genus level (Figure 2C). There were 557 unique OTUs (*Zhongshania* and *Lachnospiraceae_UCG-003*), 2,369 unique OTUs (*Clostridium_sensu_stricto_11* and *Providencia*), and 4,497 unique OTUs (*Parasegetibacter* and *Effusibacillus*) at the stages of Day 0, Day 7 and Day 28, respectively. For fungi, three stages shared 267 OTUs, mainly *Malassezia*, *Amphinema*, *Tetracladium*, *Saccharomycetales_gen_Incertae_sedis*, and *Discosia* at the genus level. There were 876 unique OTUs (*Phialocephala*), 479 unique OTUs (*Neodeightonia*), and 399 unique OTUs (*Byssochlamys*) at the stages of Day 0, Day 7 and Day 28, respectively (Figure 2D).

### Dynamics of the microbial community on the human scalp during the stages of using the fermentation filtrate from soapberry pericarp

**Figure 3:**
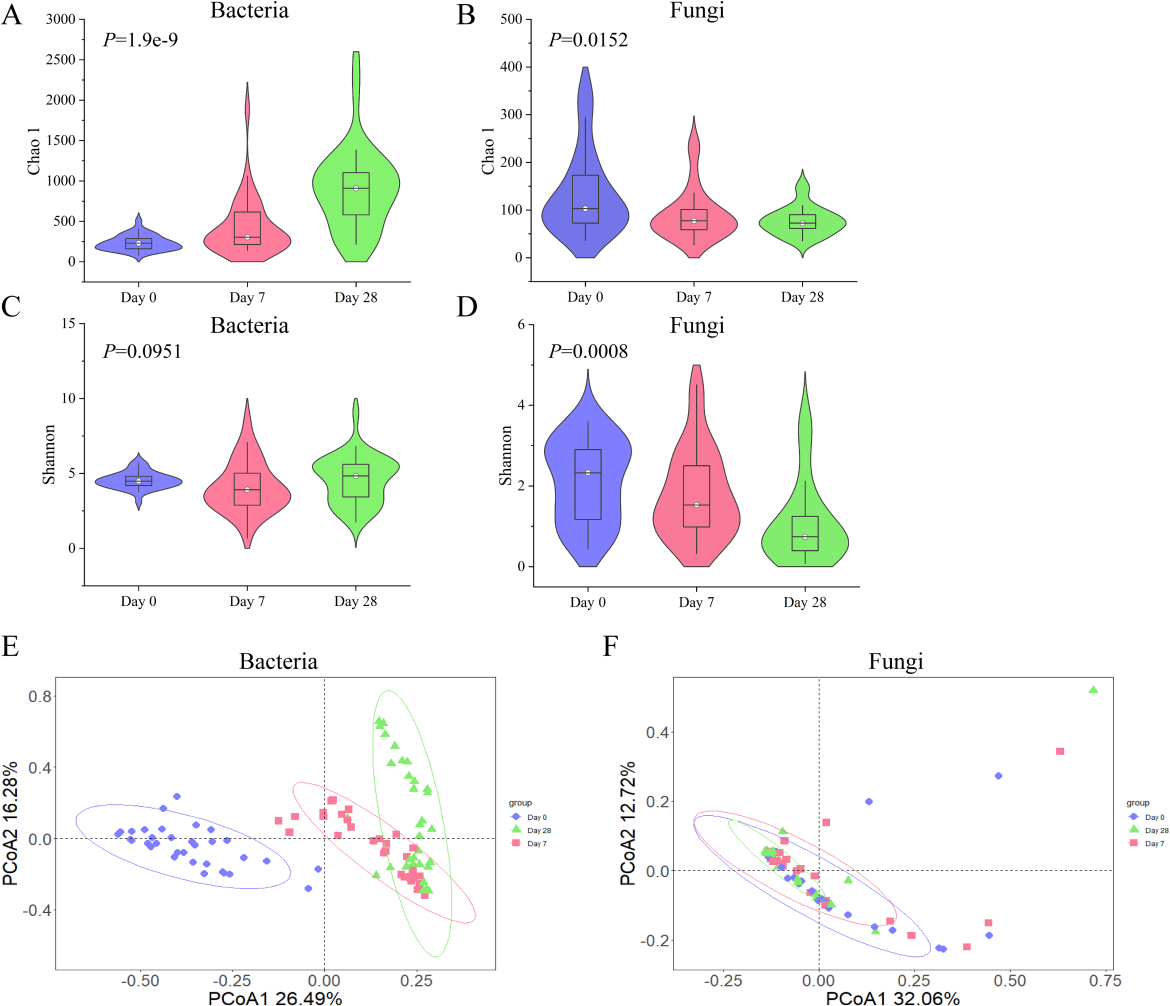
The changing trend of bacterial and fungal diversity (alpha and beta) during the stages of using the fermentation filtrate from soapberry pericarp. **(A,B)** Represent the Chao 1 index of the bacterial community and the fungal community, respectively. **(C,D)** Represent the Shannon index of the bacterial community and the fungal community, respectively. **(E,F)** Represent the Bray-Curtis distance of the bacterial community and the fungal community, respectively. Each point in the figure represents a sample, and samples of the same group are represented by the same color.

According to the stages of Day 0, Day 7, and Day 28 using the fermentation filtrate from soapberry pericarp, the microbial community composition on the human scalp varied. As shown in Figure 2, alpha (*α*)-diversity indexes were measured by Chao1 and Shannon, which represented community richness and diversity, respectively. The bacterial Chao1 index (Supplementary Table S1, Kruskal-Wallis H test, *x^2^* = 40.1299, *p* = 1.9e-9) was significantly increased in three stages. However, the fungi Chao1 index (Supplementary Table S1, Kruskal-Wallis H test, *x^2^* = 8.3737, *p* = 0.0152) significantly reduced in three stages, which was opposite to that of the bacteria (Figures 3A,B). The Shannon index, which measures both the richness and evenness of communities, showed that the changing trend of the bacteria index decreased on Day 7 samples and then increased on Day 28 samples (Supplementary Table S2, Kruskal-Wallis H test, Day 0: *M* = 4.4886, Day 7: *M* = 3.9195, Day 28: *M* = 4.8385). While the fungi showed an uninterrupted decrease (Figures 3C,D; Supplementary Table S2, Kruskal-Wallis H test, Day 0: *M* = 2.3284, Day 7: *M* = 1.5304, Day 28: *M* = 0.7407). Overall, the highest bacterial α-diversity was found in Day 28 samples when using the fermentation filtrate from soapberry pericarp, and the highest fungi α-diversity was found in Day 0 samples before using the fermentation filtrate from soapberry pericarp.

The beta (*β*)-diversity of bacterial and fungal communities at different stages of using the fermentation filtrate from soapberry pericarp was compared. It was found that the β-diversity of bacteria was significantly different at the three stages. Day 0 samples were significantly different from those of Day 7 samples (Supplementary Table S3, Adonis, Bray-Curtis, *R*^2^ = 0.26703, *p* = 0.001) and Day 28 samples (Supplementary Table S3, Adonis, Bray-Curtis, *R*^2^ = 0.37003, *p* = 0.001). Day 7 and Day 28 samples were also significantly different (Supplementary Table S3, Adonis, Bray-Curtis, *R*^2^ = 0.09509, *p* = 0.001). However, the β-diversity of fungi was significantly different between Day 0 and Day 28 samples (Supplementary Table S3, Adonis, Bray-Curtis, *R*^2^ = 0.04645, *p* = 0.019). Principal coordinate analysis (PCoA) was performed to display the results better. The Day 0, Day 7, and Day 28 samples were noticeably separated into three groups, with PCoA axis one representing 26.49% of the variation in genetic distance and PCoA axis two representing 16.28%, according to the bacterial community composition using Bray Curtis distance (Figure 3E). The Day 0, Day 7, and Day 28 samples were clustered together, and the circled area gradually decreased over time, according to the fungal community composition using Bray Curtis (Figure 3F). Overall, the results showed that the duration of using the fermentation filtrate from soapberry pericarp had a significant impact on the diversity and composition of human scalp microorganisms, especially on Day 28.

### Dynamic changes of core microbiota on the human scalp during the stages of using the fermentation filtrate from soapberry pericarp

**Figure 4:**
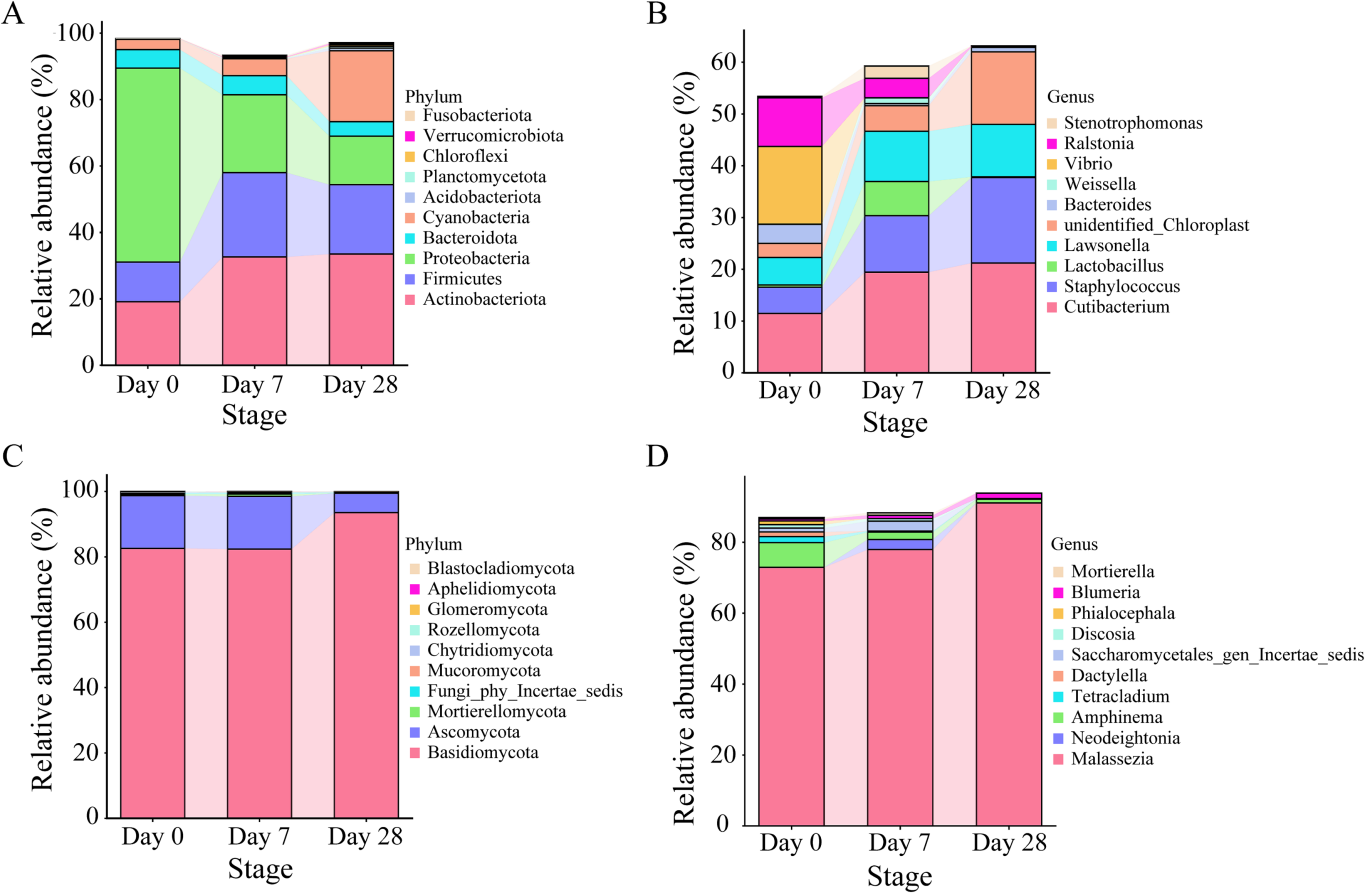
The relative abundance of core microbiota on the human scalp during the stages of using the fermentation filtrate from soapberry pericarp. **(A,B)** Represent the top 10 relative abundance of bacterial communities at the phylum and genus levels, respectively. **(C,D)** Represent the top 10 relative abundance of fungal communities at the phylum and genus levels, respectively.

In order to investigate the dynamic changes of bacterial and fungal communities on the human scalp further during the stages of using the fermentation filtrate from soapberry pericarp, phylum-, and genus-level analyses of microbial communities were performed (Figure 4). At the phylum level, *Actinobacteriota*, *Firmicutes*, *Proteobacteria*, *Bacteroidota*, and *Cyanobacteria* were the main bacteria in all stages. From Day 0 to Day 7 and Day 28, both *Actinobacteriota* and *Firmicutes* increased obviously. *Actinobacteriota* increased from 19.12 to 32.62 and 33.47% on Day 7 and Day 28, respectively, and *Firmicutes* increased from 11.97 to 25.37 and 20.91% on Day 7 and Day 28, respectively. These increases were attributed to the enrichment of the genus *Cutibacterium* (belonging to *Actinobacteriota*) and the genus *Staphylococcus* and *Lactobacillus* (belonging to *Firmicutes*) (Supplementary Tables S4, S5). Whereas the *Proteobacteria* quantities plummeted, the relative abundances decreased by 34.96% on Day 7 and 43.80% on Day 28 (Figures 4A,B). The increase was attributed to the enrichment of the genus *Vibrio* and Ralstonia (belonging to *Proteobacteria*) (Supplementary Tables S4, S5). It is noteworthy that only a few core taxa were observed or observed in high abundance in specific stages. For example, the dominant genus *Lactobacillus* accumulated in relative abundance on Day 7 and decreased dramatically on Day 28. For fungi, *Basidiomycota* and *Ascomycota* were at a similar level on Day 0 and Day 7 with high abundance, and a significant increase was seen on Day 28 (Figure 4C). At the genus level, *Malassezia* was the main fungal in all stages (Figure 4D). From Day 0 to Day 28, *Malassezia* increased dramatically from 72.93 to 91.10% (Supplementary Tables S6, S7). However, *Amphinema* existed as the second dominant genus on Day 0 and decreased on Day 7 and Day 28. Furthermore, it is interesting to note that *Neodeightonia* only appeared on Day 7 and did not appear on Day 0 and Day 28.

**Figure 5:**
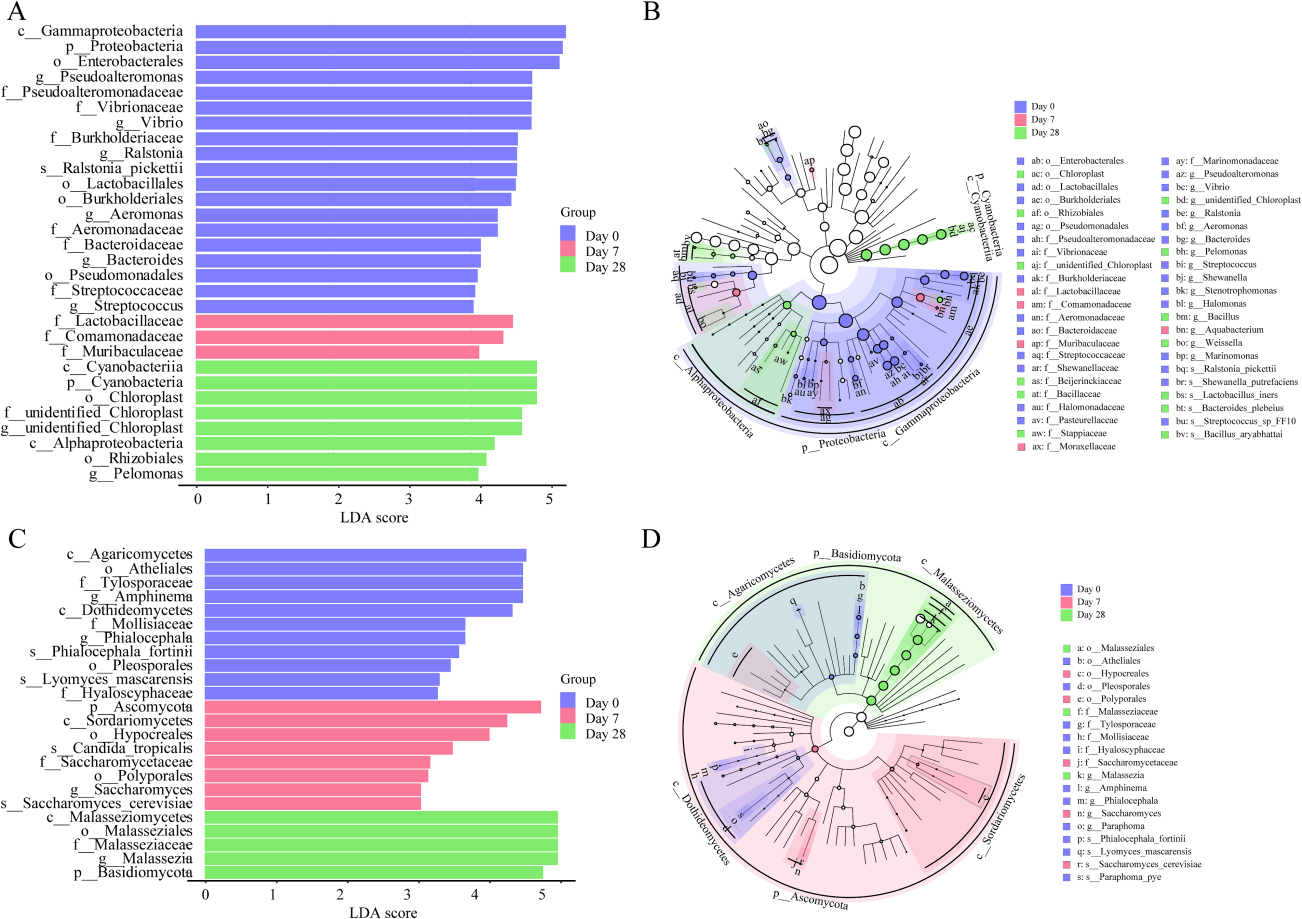
The linear discriminant analysis (LDA) and LEfSe (linear discriminant analysis effect size) were conducted based on the different operational taxonomic units (OTUs) of the human scalp microbial communities in the Day 0, Day 7 and Day 28 samples. The taxa with the highest differential abundances among the three groups were identified. **(A,C)** The LDA scores of the microbial communities were significantly different across the three groups. **(B,D)** The taxonomic cladogram was constructed by performing the LEfSe analysis. Blue, red, and green indicate taxa enriched in Day 0, Day 7, and Day 28 groups, respectively.

To further study the differences between Day 0, Day 7, and Day 28, linear discriminant analysis (LDA) effect size analysis (LEfSe) was employed. The LEfSe found 45 bacterial taxa with significant differences. A total of 27 bacterial taxa were significantly enriched on Day 0, mainly genera like *Pseudoalteromonas*, *Vibrio*, *Ralstonia*, *Aeromonas*, *Bacteroides*, and *Streptococcus*; 5 bacterial taxa were significantly enriched on Day 7, mainly families like *Lactobacillaceae* and *Comamonadaceae*; 13 bacterial taxa were significantly enriched on Day 28, mainly the genera such as *Pelomonas* (Figures 5A,B). The LEfSe found 19 fungi taxa with significant differences. 11 fungal taxa were significantly enriched on Day 0, mainly in *Amphinema* and *Phialocephala*; five fungal taxa were significantly enriched on Day 7, mainly in *Saccharomyces_cerevisiae*, *Candida_tropicalis*; and three fungal taxa were significantly enriched on Day 28, mainly in *Malassezia* (Figures 5C,D). Overall, both the bacterial and fungal communities showed significant differences on the human scalp during the stages of using the fermentation filtrate from soapberry pericarp.

### Effects of different stages of using the fermentation filtrate from soapberry pericarp on bacterial-fungal inter-kingdom networks of the scalp

**Figure 6:**
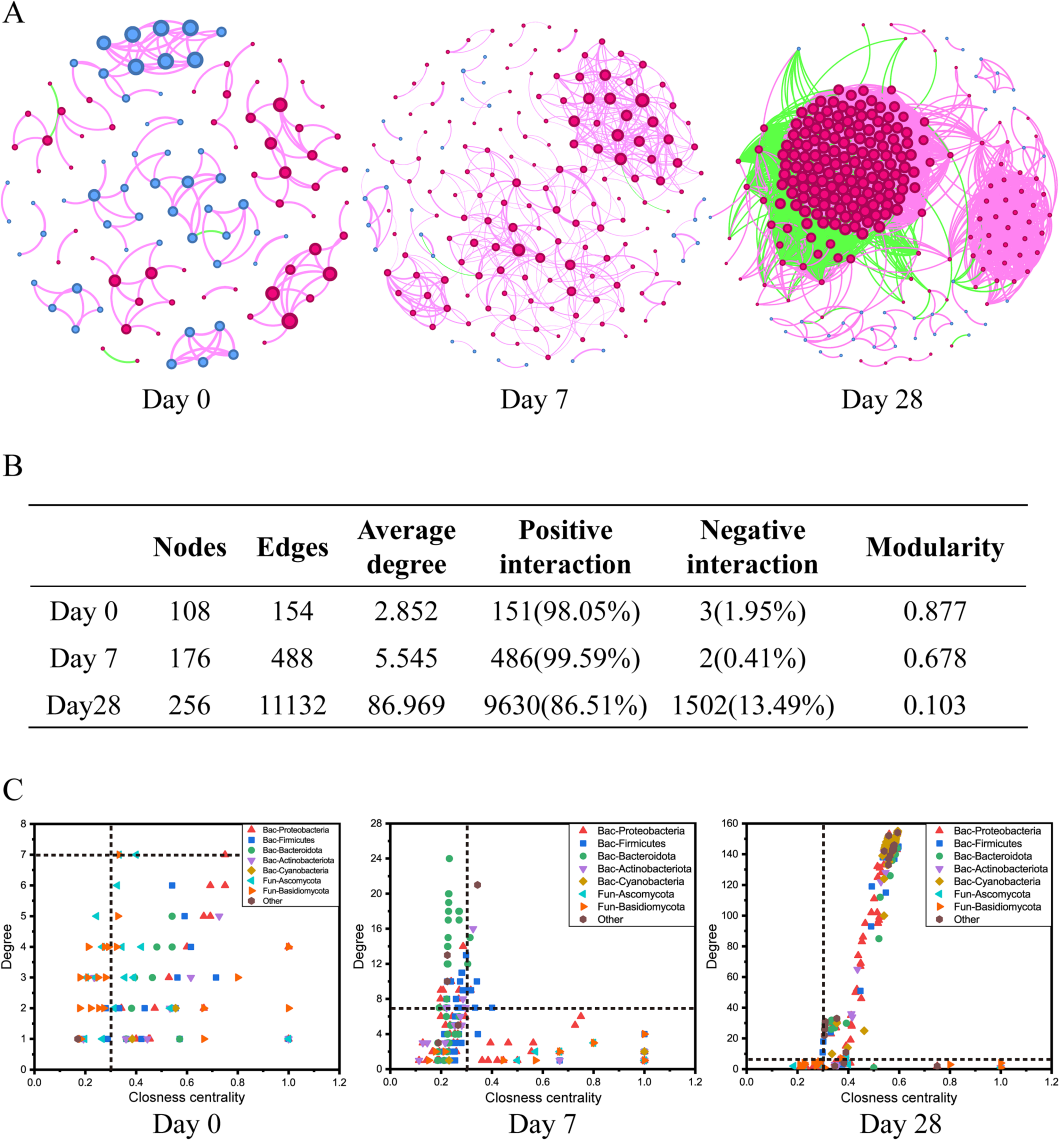
Dynamics of bacterial-fungal inter-kingdom networks during the use of the fermentation filtrate from soapberry pericarp. **(A)** Bacterial-fungal co-occurrence networks. The nodes represent the operational taxonomic units (OTUs), and the size of each node represents the degree. The red line and green line indicate positive interaction and negative interaction, respectively. **(B)** The table of topological features of bacterial-fungal inter-kingdom networks. **(C)** Changes of the topological features (degree and closeness centrality) at stages Day 0, Day 7, and Day 28.

The co-occurrence networks analysis was performed to assess the impact of the stages of using the fermentation filtrate from soapberry pericarp on bacterial-fungal inter-kingdom interactions along the human scalp. These results showed that bacterial-fungal inter-kingdom networks shifted clearly across Day 0, Day 7, and Day 28 (Figure 6). The Day 0 network (108 nodes and 154 edges) and Day 7 network (176 nodes and 488 edges) had lower average degrees than the Day 28 network (256 nodes and 11,132 edges), suggesting that the stages of using the fermentation filtrate from soapberry pericarp could increase the complexity of networks (Figures 6A,B). The order of the bacteria-fungal inter-kingdom network complexity was Day 28 > Day 7 > Day 0. Moreover, the number of positive network edges markedly increased from 151 to 9,630, and the number of negative network edges also markedly increased from 3 to 1,502 from Day 0 to Day 28. Specifically, the number of positive network edges was greater than that of negative network edges in all three stages (Figures 6A,B). This pattern indicated that the bacteria-fungi inter-kingdom network became more stable after using the fermentation filtrate from soapberry pericarp.

When the node degree value was greater than or equal to 7, and the closeness centrality was greater than 0.3 as the screening criterion, there were seven key species (bacteria 1, fungi 6), nine key species (bacteria 9, fungi 0), and 198 key species (bacteria 195, fungi 3) at the stages Day 0, Day 7, and Day 28, respectively (Figure 6C and Supplementary Figure S1). Among them, bacterial nodes were mainly in *Proteobacteria*, *Firmicutes*, *Bacteroidota*, *Actinobacteria,* and *Cyanobacteria*, and fungal nodes were mainly in *Ascomycota* and *Basidiomycota* at the phylum level (Figure 6C). The differences in co-occurrence networks were further analyzed by calculating the number of common and unique nodes among the stages Day 0, Day 7, and Day 28. Compared with Day 0 and Day 7, Day 28 shared one node and three nodes with Day 0 and Day 7, respectively, and had 194 unique nodes (Supplementary Figure S1). Thus, using the fermentation filtrate from soapberry pericarp could form a unique microbial co-occurrence network.

### Predicted functional profiling of the scalp microbiome through PICRUSt2 and FUNGuild

**Figure 7:**
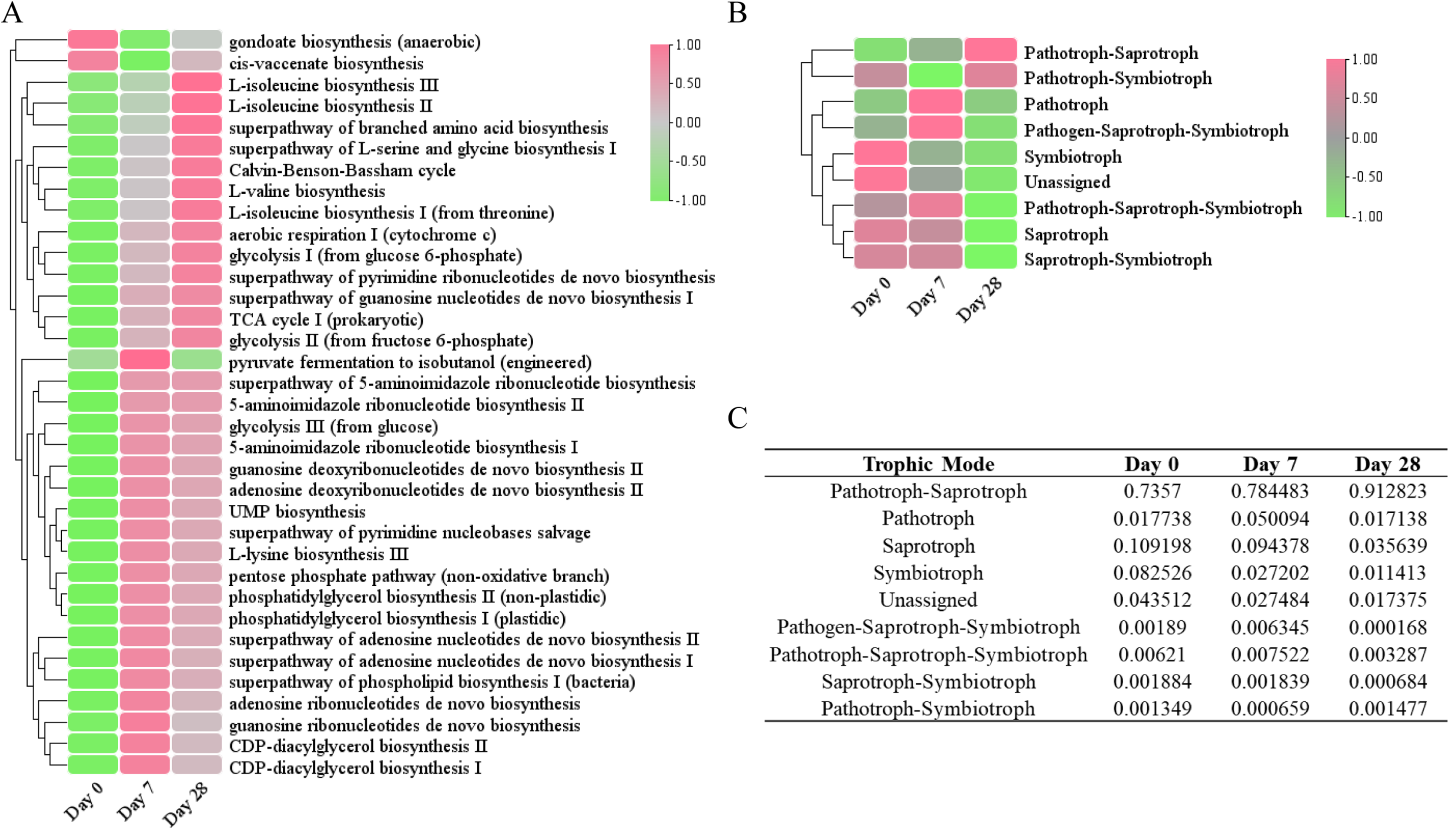
Functional annotation of the human scalp microbial communities during the stages of using the fermentation filtrate from soapberry pericarp. **(A)** PICRUSt2 functional prediction (bacterial). **(B)** FUNGuild functional prediction (fungal). **(C)** The table of relative abundances of fungal OTUs classified by trophic mode.

To better understand the role of the fermentation filtrate from soapberry pericarp in the scalp microbiome, we predicted the function of bacterial and fungal microbiota in the samples. Based on the PICRUSt2 results, we identified 451 unique MetaCyc pathways and displayed the top 35 functional pathways with relatively high abundance using a heat map (Figure 7A). The predicted functional analysis in all three stages found mainly affiliations with the biosynthesis of amino acids, nucleosides, nucleotides, fatty acids, and lipids, as well as the generation of precursor metabolites and energy. Among the MetaCyc ontology classifications, gondoate biosynthesis (anaerobic) and cis-vaccenate biosynthesis were the most abundant on Day 0, whereas the other pathways were noticeably lower on Day 0 compared to other groups, indicating the biosynthesis of fatty acid and lipid were downregulated on Day 7 and Day 28, the biosynthesis of amino acid, nucleoside, and nucleotide, and the generation of precursor metabolites and energy were upregulated on Day 7 and Day 28 (Figure 7A and Supplementary Table S8).

Furthermore, the fungal community functions of the three stages were also predicted (Figure 7B). Most of the fungal trophic modes identified on Day 0 were pathotroph-saprotroph (73.57%), followed by saprotroph (10.92%), and symbiotroph (8.25%) (Figures 7B,C). During the stages of using the fermentation filtrate from soapberry pericarp, pathotroph-saprotroph fungi (animal pathogen-undefined saprotroph) increased on Day 7 (78.45%) and continued to increase on Day 28 (91.28%); however, saprotroph (undefined saprotroph and dung saprotroph-undefined saprotroph-wood saprotroph) and symbiotroph (ectomycorrhizal and endophyte) relative abundance declined consistently throughout. Overall, the fermentation filtrate from soapberry pericarp altered the function of the fungal community on the human scalp.

## Discussion

This study provided the first systematic analysis of how the microorganisms on the human scalp change dynamically during the stages of using the fermentation filtrate from soapberry pericarp. The relationship between the microbial and used stages was captured, thereby increasing the understanding of the fermentation filtrate of the soapberry pericarp-associated microbial community.

### Changes in microbial diversity during the stages of using the fermentation filtrate from soapberry pericarp

The data showed that the microbial communities on the human scalp were not static patterns but formed dynamic changes with the stages of using the fermentation filtrate from soapberry pericarp. Based on the Chao 1 index, bacteria and fungi showed significant differences in this study, implying that the richness of the microbial community has already changed. For the bacteria community, the Chao 1 index exhibited a significant increase, potentially attributable to the presence of numerous bioactive compounds in the fermentation filtrate from soapberry pericarp, including saponins and amino acids, which may serve as nutrient sources for microorganisms, thereby promoting bacterial growth and reproduction. Conversely, the Chao 1 index demonstrated a significant decrease for the fungi community. This reduction could be due to the effective washing properties of the soapberry pericarp fermentation filtrate, which removes grease soiling from the human scalp. Since sebum constitutes a crucial nutrient source for the growth of most fungi on the scalp, particularly *Malassezia*, its removal likely impedes fungal proliferation (Xu et al., 2016). Based on the Shannon index, the microbial *α*-diversity of bacteria showed no significant differences in this study, indicating that the composition of the bacterial community remained unchanged. However, the microbial α-diversity of fungal showed significant differences in this study (Day 0 > Day 7 > Day 28), indicating that the fungal communities had an overall downward trend during the stages of using the fermentation filtrate from soapberry pericarp, which is in contrast to the results of Saxena et al. (2021). This study found that the α-diversity (Shannon index) significantly increased in the bacterial and fungal microbiome when coconut oil was topically applied to a healthy scalp (Saxena et al., 2021). This phenomenon can be attributed to our project recruiting participants with Chinese scalps rather than Indian scalps.

In general, the dominant phyla on the human scalp were *Actinobacteriota* and *Firmicutes*, and the dominant genera were *Cutibacterium* and *Staphylococcus* (Grimshaw et al., 2019; Won et al., 2022; Tsai et al., 2023), which is consistent with the findings obtained in this study. Notably, this study observed a higher relative abundance of *Actinobacteria* than *Firmicutes*. A potential explanation for this observation is that *Actinobacteria* possess the ability to metabolize a diverse array of organic compounds, including grease and dandruff, which facilitates their growth and reproduction (Dhanasekaran and Jiang, 2016; Li et al., 2022). In contrast, many members of the phylum *Firmicutes* are anaerobic or facultatively anaerobic bacteria. Given that the scalp surface is relatively exposed and has a high oxygen content, the growth of *Firmicutes* may be inhibited. Additionally, *Actinobacteria* are capable of producing antibiotics that suppress the proliferation of various other bacterial species (Zothanpuia et al., 2018). Consequently, *Actinobacteria* exhibit a competitive advantage over *Firmicutes* in this ecological niche.

It is worth noting that 16S rRNA amplicon analysis is a commonly used method in microbiome composition research and is often utilized to elucidate the scalp microbiome. It provides reliable genus-level classification, but it has limited species-level discrimination ability. The results suggested that the role of fermentation filtrate of soapberry pericarp could regulate the proportion of microbial communities, which contribute to the health of scalp microorganisms. The mode of action involves the activity of bioactive compounds. Compounds 4, 5, 6, 7, 8, 9, 10, 11 and 12 are glycolipids among the most popular biosurfactants. Based on their chemical structure, glycolipids are constituted by a fatty acid in combination with a carbohydrate moiety and correspond to a group of compounds that differ by the nature of the lipid and carbohydrate moiety (Ines and Dhouha, 2015). Compounds 13, 14, 15, 16, 17, and 18 are triterpenoid saponins with two basic structural units: glycone and aglycone. The glycone unit functions as a hydrophilic moiety, and the aglycone unit functions as a lipophilic moiety, responsible for triterpenoid saponins’ distinctive characteristics and biological activities (Jolly et al., 2024). In this study, the regulation of fermentation filtrate of soapberry pericarp on human scalp microorganisms may be accompanied by triterpenoid saponins, which affect the formation of biofilm and function as a surfactant. Compound 19 is reported to function in cosmetics as a hair conditioning agent and as a surfactant, a cleansing agent (Fiume et al., 2021; Schreiner et al., 2022). Therefore, this study could serve as the basis for understanding the relationship between the fermentation filtrate from soapberry pericarp and scalp microbiome and pave the way for its application in healthy products.

### Microbial co-occurrence network in different stages of the fermentation filtrate from soapberry pericarp

Like plant and soil microbial communities, human scalp microbial communities also have many complex negative or positive interactions, such as competition, parasitism, and symbiosis (de Menezes et al., 2017; Huo et al., 2023). The inter-kingdom co-occurrence network is a good approach to revealing the dynamics of microbial associations during the stages of using the fermentation filtrate from soapberry pericarp. The most complicated network was at stage Day 28, suggesting the fermentation filtrate from soapberry pericarp could increase interactions among microbial community members (Figure 6). This trend in the increasing complexity of the microbial community was likely due to the fermentation filtrate from soapberry pericarp influencing the microbiota composition by providing a few nutrients, such as amino acids and polysaccharides. The complexity of the microbial network is closely correlated with its stability, and the stability of the network rises with network complexity, which is congruent with the ecological view (Mougi and Kondoh, 2012; Ma et al., 2022; Liu et al., 2024). This result implies that the use of fermentation filtrate of soapberry pericarp increased the stability of the human scalp microbial community structure and, therefore, improved the ability to resist external interference. In addition, the stages Day 0 and Day 7 of the inter-kingdom co-occurrence network showed a high degree of modularity and fewer nodes and edges, while the stage Day 28 showed a low degree of modularity and more nodes and edges (Figures 6A,B).

High modularity values represent a high degree of niche differentiation, which may lead to weakened microbial interactions (Faust and Raes, 2012). This explains why the number of connections at the stages Day 0 and Day 7 is lower. Thus, the use of fermentation filtrate of soapberry pericarp was likely conducive to increasing microbial interactions and helping improve microbial community stability.

### The effect of fermentation filtrate of soapberry pericarp on microbial community function

The scalp can secrete an appropriate amount of oil so that the hair is moisturized and has a lustrous appearance. However, excessive oil secretion may disrupt the skin barrier of the scalp, leading to various scalp problems such as itching, dandruff, folliculitis, and scalp hair loss (DeAngelis et al., 2005; Patel et al., 2017; Polak-Witka et al., 2020). Moreover, the results have shown that the pathways related to vitamins and coenzyme factors (such as metabolism of biotin, chlorophyll, vitamin B6, niacin and niacinamide, biosynthesis of ubiquinone and other terpenoquinones), amino acids (histidine, cysteine, and methionine metabolism), and lipoic acid are abundant in a healthy scalp (Nisenson, 1969; Grafe et al., 2003; Leblanc et al., 2013; Uchida et al., 2015; Biagi et al., 2016; Patel et al., 2017; Saxena et al., 2018).

In the present study, it was found that the biosynthesis of fatty acids and lipids could be regulated after the use of fermentation filtrate from soapberry pericarp. The biosynthesis of gondoate (anaerobic) and cis-vaccinate was downregulated compared to Day 0 (Figure 7A), which may suggest that the fermentation filtrate from soapberry pericarp may have a beneficial effect on people with excessive oil secretion. It was also found that the biosynthesis of amino acids (L-isoleucine, L-lysine, L-valine, L-serine, and glycine), nucleosides and nucleotides (such as 5-aminoimidazole ribonucleotides, UMP, pyrimidine ribonucleotides, and guanosine nucleotides *de novo*), as well as the generation of precursor metabolites and energy (such as TCA cycle, glycolysis pentose phosphate pathway), were enriched after the use of fermentation filtrate of soapberry pericarp (Figure 7A). Among the amino acids, the lysine amino acid is an essential amino acid known to restore scalp condition and reduce hair loss, while L-serine and glycine are known to improve the water-holding capacity of the skin. The branched-chain amino acids (L-isoleucine and L-valine) are not only the basic units of protein composition but also regulate protein translation to increase protein synthesis (Rushton, 2002; Zhang et al., 2017; Liuqin et al., 2020). Taken together, the above results indicate that the fermentation filtrate from soapberry pericarp could improve human scalp health. To deeply understand the effect of fermentation filtrate of soapberry pericarp on scalp microbiota, the changes in the physical properties of the scalp must be further studied.

## Conclusion

The study demonstrated that the application of the fermentation filtrate from soapberry pericarp on the scalp affected microbial diversity and composition. This change was mainly associated with the use stage of the fermentation filtrate from soapberry pericarp. The human scalp hosts different microbes at various stages, but the core community remains. Dominant microbes include *Actinobacteriota*, *Firmicutes*, and *Proteobacteria*. From Day 0 to Day 7 and Day 28, *Actinobacteriota* and *Firmicutes* increased, while *Proteobacteria* decreased, promoting healthier scalp microorganisms. In addition, the predicted functional analysis for microbial communities suggested that the fermentation filtrate from soapberry pericarp may improve human scalp health by controlling oil secretion and regulating the biosynthesis of vitamins, amino acids, and so on. It will help rationally utilize the fermentation filtrate from soapberry pericarp to maintain or improve human scalp health.

## Data Availability

The original contributions presented in the study are included in the article/Supplementary material, further inquiries can be directed to the corresponding author.

## Footnotes

^1^http://ccb.jhu.edu/software/FLASH/

^2^http://drive5.com/uparse/

^3^http://www.arb-silva.de/

^4^https://unite.ut.ee/

^5^https://www.home-for-researchers.com/#/statistics/metering_more

^6^https://magic.novogene.com
