# Supplementary material for: Investigation of the fermentation filtrate from soapberry (Sapindus mukorossi Gaertn.) pericarp on improving the microbial diversity and composition of the human scalp
Source: Front Microbiol. 2024 Oct 10;15:1443767. doi: 10.3389/fmicb.2024.1443767 (PMC11499179; doi:10.3389/fmicb.2024.1443767)
Supplement: Supplementary file 1 [file Data_Sheet_1.docx]

**Supporting information**

**Investigation of the Fermentation Filtrate of Soapberry (*Sapindus mukorossi* Gaertn.) pericarp on improving the diversity and microbial composition on Human Scalp**

Chong Xu^1^, Danyang Pan^1^, Dexiang Zhang^1^, Lin Lin^1^, Yiti Cheng^1^, Shuangcheng Liang^2^, Jingyu He^1,3^*


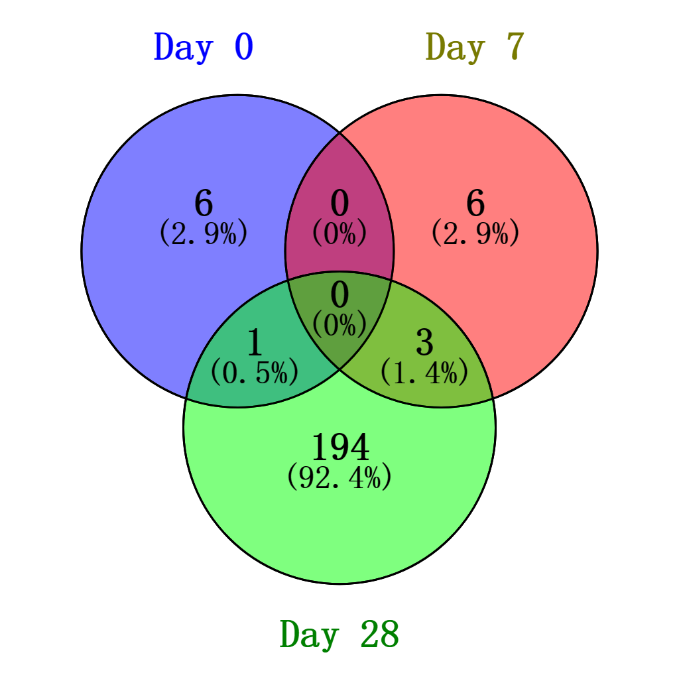


**Figure S1** The wayne diagram of the nodes in bacterial-fungal inter-kingdom networks among the Day 0, Day 7 and Day 28

**Table S1** Kruskal-Wallis H test on Chao1 index in Day 0, Day 7, and Day 28 groups.

| Group | Bacterial | | | Fungal | | |
| --- | --- | --- | --- | --- | --- | --- |
|  | *M* | *X^2^* | *P* | *M* | *X^2^* | *P* |
| Day 0 | 230 | 40.1299 | **1.9e-9** | 103 | 8.3737 | **0.0152** |
| Day 7 | 304.0333 |  |  | 77.5 |  |  |
| Day 28 | 910.0255 |  |  | 72.5455 |  |  |

Bold values indicate statistically significant results, *P* < 0.05.

**Table S2** Kruskal-Wallis H test on Shannon index in Day 0, Day 7, and Day 28 groups.

| Group | Bacterial | | | Fungal | | |
| --- | --- | --- | --- | --- | --- | --- |
|  | *M* | *X^2^* | *P* | *M* | *X^2^* | *P* |
| Day 0 | 4.4886 | 4.7051 | 0.0951 | 2.3284 | 14.2848 | **0.0008** |
| Day 7 | 3.9195 |  |  | 1.5304 |  |  |
| Day 28 | 4.8385 |  |  | 0.7407 |  |  |

Bold values indicate statistically significant results, *P* < 0.05.

**Table S3** Adonis test based on bray-curtis distances.

| Group | Bacterial | | Fungal | |
| --- | --- | --- | --- | --- |
|  | R^2^ | *P* | R^2^ | *P* |
| Day 0 vs Day 7 | 0.26703 | **0.001** | 0.02813 | 0.125 |
| Day 0 vs Day 28 | 0.37003 | **0.001** | 0.04645 | **0.019** |
| Day 7 vs Day 28 | 0.09509 | **0.001** | 0.03255 | 0.108 |

Bold values indicate statistically significant results, *P* < 0.05.

**Table S4** Relative abundance of top 10 dominant bacterial phylum in different group

| Taxonomy | Actinobacteriota | Firmicutes | Proteobacteria | Bacteroidota | Cyanobacteria | Acidobacteriota | Planctomycetota | Chloroflexi | Verrucomicrobiota | Fusobacteriota |
| --- | --- | --- | --- | --- | --- | --- | --- | --- | --- | --- |
| Day 0 | 19.1060% | 11.9724% | 58.4029% | 5.5543% | 3.0851% | 0.0511% | 0.0456% | 0.0967% | 0.0520% | 0.0973% |
| Day 7 | 32.6196% | 25.3743% | 23.4439% | 5.7692% | 5.0975% | 0.1598% | 0.1773% | 0.1330% | 0.1979% | 0.3829% |
| Day 28 | 33.4694% | 20.9108% | 14.6022% | 4.3479% | 21.3889% | 0.7566% | 0.5578% | 0.4543% | 0.4115% | 0.2427% |

**Table S5** Relative abundance of top 10 dominant bacterial genus in different group

| Taxonomy | Cutibacterium | Staphylococcus | Lactobacillus | Lawsonella | unidentified_Chloroplast | Bacteroides | Weissella | Vibrio | Ralstonia | Stenotrophomonas |
| --- | --- | --- | --- | --- | --- | --- | --- | --- | --- | --- |
| Day 0 | 11.4757% | 5.0710% | 0.4334% | 5.2962% | 2.7307% | 3.6782% | 0.0258% | 15.0025% | 9.4183% | 0.2696% |
| Day 7 | 19.4496% | 10.9080% | 6.5937% | 9.7064% | 4.9445% | 0.4024% | 1.1019% | 0.0284% | 3.7560% | 2.3643% |
| Day 28 | 21.2079% | 16.5266% | 0.1290% | 10.1295% | 14.0191% | 0.8564% | 0.0653% | 0.0913% | 0.0956% | 0.0337% |

**Table S6** Relative abundance of top 10 dominant fungal phylum in different group

| Taxonomy | Basidiomycota | Ascomycota | Mortierellomycota | Fungi_phy_Incertae_sedis | Mucoromycota | Chytridiomycota | Rozellomycota | Glomeromycota | Aphelidiomycota | Blastocladiomycota |
| --- | --- | --- | --- | --- | --- | --- | --- | --- | --- | --- |
| Day 0 | 82.5872% | 16.1559% | 0.4468% | 0.4291% | 0.0298% | 0.2366% | 0.0653% | 0.0149% | 0.0013% | 0.0180% |
| Day 7 | 82.3684% | 16.1217% | 0.7245% | 0.4526% | 0.0511% | 0.0862% | 0.0913% | 0.0639% | 0.0239% | 0.0015% |
| Day 28 | 93.5162% | 5.9339% | 0.0246% | 0.2495% | 0.1312% | 0.0098% | 0.0829% | 0.0371% | 0.0004% | 0.0141% |

**Table S7**Relative abundance of top 10 dominant fungal genus in different group

| Taxonomy | Malassezia | Neodeightonia | Amphinema | Tetracladium | Dactylella | Saccharomycetales_gen_Incertae_sedis | Discosia | Phialocephala | Blumeria | Mortierella |
| --- | --- | --- | --- | --- | --- | --- | --- | --- | --- | --- |
| Day 0 | 72.9323% | 0% | 6.9494% | 1.6997% | 1.3358% | 1.0733% | 0.9772% | 1.0368% | 0.5345% | 0.4262% |
| Day 7 | 77.9543% | 2.8354% | 2.0717% | 0.2945% | 0% | 2.7923% | 0.7280% | 0% | 0.9262% | 0.6957% |
| Day 28 | 91.0989% | 0% | 1.0372% | 0.0195% | 0.0029% | 0.1547% | 0.0019% | 0% | 1.5375% | 0.0246% |

**Table S8**The table displays the relative pathway of bacterial prediction by PICRUSt2

| **Pathway** | **Description** | **Associated biosynthesis pathways** |
| --- | --- | --- |
| PWY-7663 | gondoate biosynthesis (anaerobic) | Fatty acid biosynthesis |
| PWY-5973 | cis-vaccenate biosynthesis |  |
| PWY-5103 | L-isoleucine biosynthesis III | Amino acid biosynthesis |
| PWY-5101 | L-isoleucine biosynthesis II |  |
| BRANCHED-CHAIN-AA-SYN-PWY | superpathway of branched amino acid biosynthesis |  |
| SER-GLYSYN-PWY | superpathway of L-serine and glycine biosynthesis I |  |
| CALVIN-PWY | Calvin-Benson-Bassham cycle | Carbon consumption |
| VALSYN-PWY | L-valine biosynthesis | Amino acid biosynthesis |
| ILEUSYN-PWY | L-isoleucine biosynthesis I (from threonine) |  |
| PWY-3781 | aerobic respiration I (cytochrome c) | Generation of precursor metabolites and energy |
| GLYCOLYSIS | glycolysis I (from glucose 6-phosphate) |  |
| PWY0-162 | superpathway of pyrimidine ribonucleotides de novo biosynthesis | Nucleoside and Nucleotide Biosynthesis |
| PWY-7228 | superpathway of guanosine nucleotides de novo biosynthesis I |  |
| TCA | TCA cycle I (prokaryotic) | Generation of precursor metabolites and energy |
| PWY-5484 | glycolysis II (from fructose 6-phosphate) |  |
| PWY-7111 | pyruvate fermentation to isobutanol (engineered) |  |
| PWY-6277 | superpathway of 5-aminoimidazole ribonucleotide biosynthesis | Nucleoside and nucleotide biosynthesis |
| PWY-6122 | 5-aminoimidazole ribonucleotide biosynthesis II |  |
| ANAGLYCOLYSIS-PWY | glycolysis III (from glucose) | Generation of precursor metabolites and energy |
| PWY-6121 | 5-aminoimidazole ribonucleotide biosynthesis I | Nucleoside and nucleotide biosynthesis |
| PWY-7222 | guanosine deoxyribonucleotides de novo biosynthesis II |  |
| PWY-7220 | adenosine deoxyribonucleotides de novo biosynthesis II |  |
| PWY-5686 | UMP biosynthesis |  |
| PWY-7208 | superpathway of pyrimidine nucleobases salvage |  |
| PWY-2942 | L-lysine biosynthesis III | Amino acid biosynthesis |
| NONOXIPENT-PWY | pentose phosphate pathway (non-oxidative branch) | Generation of precursor metabolites and energy |
| PWY4FS-8 | phosphatidylglycerol biosynthesis II (non-plastidic) | Fatty acid and lipid biosynthesis |
| PWY4FS-7 | phosphatidylglycerol biosynthesis I (plastidic) |  |
| PWY-6126 | superpathway of adenosine nucleotides de novo biosynthesis II | Nucleoside and nucleotide biosynthesis |
| PWY-7229 | superpathway of adenosine nucleotides de novo biosynthesis I |  |
| PHOSLIPSYN-PWY | superpathway of phospholipid biosynthesis I (bacteria) | Fatty acid and lipid biosynthesis |
| PWY-7219 | adenosine ribonucleotides de novo biosynthesis | Nucleoside and nucleotide biosynthesis |
| PWY-7221 | guanosine ribonucleotides de novo biosynthesis |  |
| PWY0-1319 | CDP-diacylglycerol biosynthesis II | Phospholipid biosynthesis |
| PWY-5667 | CDP-diacylglycerol biosynthesis I |  |
